# Interactions of 7,8-Dihydroxyflavone with Serum Albumin as well as with CYP2C9, CYP2C19, CYP3A4, and Xanthine Oxidase Biotransformation Enzymes

**DOI:** 10.3390/biom9110655

**Published:** 2019-10-25

**Authors:** Eszter Fliszár-Nyúl, Violetta Mohos, Tímea Bencsik, Beáta Lemli, Sándor Kunsági-Máté, Miklós Poór

**Affiliations:** 1 Department of Pharmacology, Faculty of Pharmacy, University of Pécs, Szigeti út 12, H-7624 Pécs, Hungary; eszter.nyul@aok.pte.hu (E.F.-N.); mohos.violetta@gytk.pte.hu (V.M.); 2 János Szentágothai Research Centre, University of Pécs, Ifjúság útja 20, H-7624 Pécs, Hungary; beata.lemli@aok.pte.hu (B.L.); kunsagi-mate.sandor@gytk.pte.hu (S.K.-M.); 3 Department of Pharmacognosy, Faculty of Pharmacy, University of Pécs, Rókus u. 2, H-7624 Pécs, Hungary; timea.bencsik@aok.pte.hu; 4 Institute of Organic and Medicinal Chemistry, Medical School, University of Pécs, Szigeti út 12, H-7624 Pécs, Hungary

**Keywords:** 7,8-dihydroxyflavone, serum albumin, cytochrome P450 enzymes, xanthine oxidase, pharmacokinetic interactions

## Abstract

7,8-dihydroxyflavone (DHF) is a flavone aglycone which has beneficial effects in several central nervous system diseases. Most of the pharmacokinetic properties of DHF have been characterized, while only limited information is available regarding its interactions with serum albumin and biotransformation enzymes. In this study, the interactions of DHF with albumin was examined employing fluorescence spectroscopy and ultrafiltration. Furthermore, the inhibitory effects of DHF on cytochrome P450 (CYP2C9, CYP2C19, and CYP3A4) and xanthine oxidase (XO) enzymes were also tested using in vitro models. Our results demonstrate that DHF forms a stable complex with albumin (*K* = 4.9 × 10^5^ L/mol) and that it is able to displace both Site I and Site II ligands. Moreover, DHF proved to be a potent inhibitor of each enzyme tested, showing similar or slightly weaker effects than the positive controls used. Considering the above-listed observations, the coadministration of DHF with drugs may interfere with the drug therapy due to the development of pharmacokinetic interactions.

## 1. Introduction

Flavonoids are ubiquitous phenolic compounds in nature, and they exert several beneficial effects [1]. Furthermore, flavonoids can affect several proteins, including numerous enzymes, transporters, and receptors [2,3]. Due to their extensive presystemic elimination, the normal dietary intake of flavonoids results in only their nanomolar concentrations in the systemic circulation [4]. However, as a result of extremely high intake (e.g., through the consumption of dietary supplements or medications), the plasma concentrations of flavonoids (or some of their metabolites) can achieve micromolar levels [5,6]. Depending on their structures, flavonoids can inhibit or induce different biotransformation enzymes [7,8]. Therefore, the coadministration of flavonoids with drugs may interfere with the drug therapy [9].

**Figure 1:**
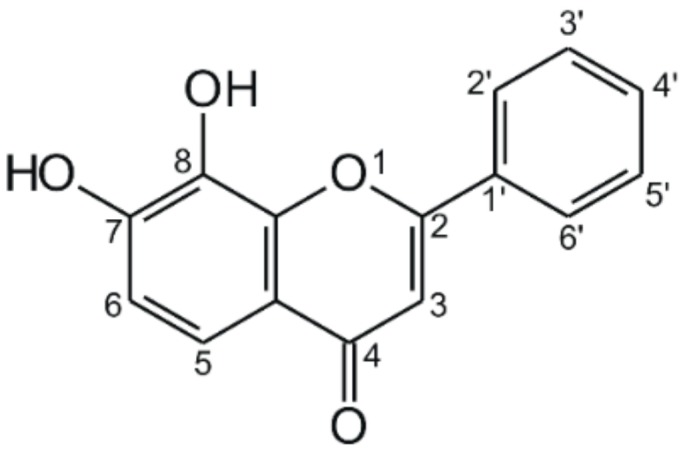
Chemical structure of 7,8-dihydroxyflavone.

7,8-dihydroxyflavone (DHF; Figure 1) is a flavone aglycone; it occurs in *Tridax procumbens* (Asteraceae) and *Godmania aesculifolia* (Bignoniaceae) [10]. Due to its ability to penetrate into the central nervous system, DHF can act as a tyrosine kinase receptor B (TrkB) agonist in the brain; therefore, it mimics the activity of brain-derived neurotrophic factor (BDNF) [10,11]. Based on recent studies, DHF is a promising candidate for the treatment of neurodegenerative diseases, including neurodevelopmental disorders, depression, Rett syndrome, and Alzheimer’s disease [3,11,12]. Furthermore, DHF is able to reverse the long-term central nervous system effects of chronic lead exposure in children due to its BDNF mimetic activity [13]. Despite the fact that DHF has better pharmacokinetic properties (e.g., better oral bioavailability and longer elimination half-life) than orally administered BDNF, it still has a poor oral bioavailability (approximately 4.5% in mice) [14], which can be improved 2.3-fold by 7,8-bis-carbamate ester prodrug formation [14]. During its biotransformation, DHF undergoes conjugation reactions; primarily glucuronide and sulfate metabolites were formed in monkeys after the per os administration of 30 mg/kg DHF [15].

Human serum albumin (HSA) is the most abundant plasma protein in human circulation. HSA is a carrier of numerous endogenous and exogenous compounds in the blood, including fatty acids, drugs, and toxins [16]. There are two major drug-binding sites of drugs in HSA, which are located in subdomains IIA (Sudlow’s Site I) and IIIA (Sudlow’s Site II). Bulky, heterocyclic compounds with negative delocalized charge are the typical ligands of Site I, while aromatic carboxylic acids preferably bind to Site II [16]. Plasma protein binding can affect the pharmacokinetics and pharmacodynamics of drugs because only unbound drug molecules are able to leave the circulation and to produce pharmacological effects in other tissues [17]. Therefore, the displacement of strongly albumin-bound drugs from HSA can significantly elevate their unbound concentration, which can increase their therapeutic effects and/or may lead to the development of side effects or even toxicity [16,17]. Simultaneous administration of two or more drugs with high affinity to HSA may result in displacement interaction, which can be caused by either allosteric or competitive mechanisms [16].

Cytochrome P450 (CYP) enzymes are responsible for the biotransformation of several exogenous and endogenous compounds. CYP3A4 metabolizes approximately 50% of medications, including several immunosuppressants, anticancer drugs, statins, calcium channel blockers, and steroid hormones [18]. CYP2C9 plays an important role in the biotransformation of certain oral anticoagulants, antidiabetics, and nonsteroidal anti-inflammatory drugs [19]. CYP2C19 catalyzes the biotransformation of some antiepileptic drugs, proton pump inhibitors, and tricyclic antidepressants [20,21].

Xanthine oxidase (XO) is a flavoprotein enzyme which plays a key role in the oxidation of hypoxanthine to xanthine and then to uric acid. Physiologically, it exists in xanthine dehydrogenase form, which can be converted to XO by proteolysis or oxidation in response to global ischaemia [22]. Therefore, XO is associated with the development of several pathological conditions such as inflammation, gout, metabolic disorders, and carcinogenesis due to the formation of uric acid and reactive superoxide anion radicals [23,24].

DHF is a promising candidate for the therapy of neurodegenerative disorders but only limited information is available regarding its albumin binding and effects on biotransformation enzymes. These properties play important roles in the development of potential pharmacokinetic interactions. Therefore, in this study, the interactions of DHF with HSA as well as with CYP2C9, CYP2C19, CYP3A4, and XO enzymes were investigated. The complex formation of DHF with HSA was examined employing fluorescence quenching. Displacement of site markers from HSA by DHF were tested using ultrafiltration. To test the inhibitory effects of DHF on biotransformation enzymes, in vitro enzyme assays were performed. Our results suggest that DHF may interfere with the albumin binding and/or the biotransformation of several dugs, suggesting the potential importance of its pharmacokinetic interactions.

## 2. Materials and Methods

### 2.1. Reagents

7,8-dihydroxyflavone (DHF) was obtained from Extrasynthese (Genay Cedex, France). *S*-mephenytoin, 4-hydroxymephenytoin, diclofenac, 4′-hydroxydiclofenac, and 6-thiouric acid were purchased from Carbosynth (Berkshire, UK). High-performance liquid chromatography (HPLC) grade acetonitrile and methanol were acquired from VWR (Budapest, Hungary). Orthophosphoric acid (85 *v/v*%), human serum albumin (HSA), racemic warfarin (WAR), naproxen (NAP), testosterone, 6β-hydroxytestosterone, naringenin, ticlopidine hydrochloride, 6-mercaptopurin, allopurinol, CypExpress 2C9 kit, CypExpress 2C19 kit, CypExpress 3A4 kit, and xanthine oxidase (XO; from bovine milk) were obtained from Sigma-Aldrich (Budapest, Hungary). Nicotinamide adenine dinucleotide phosphate sodium salt (NADP^+^) and glucose-6-phosphate barium salt (G6P) were purchased from Reanal (Budapest, Hungary). Glacial acetic acid (99.5 *v/v*%) was obtained from Fluka (Bucharest, Romania). Stock solutions of DHF, site markers, substrates, and products (2000 μM each) were prepared in dimethyl sulfoxide (spectroscopic grade; Fluka) and stored at −20°C.

### 2.2. Spectroscopic Measurements

Fluorescence spectroscopic measurements were carried out using a Hitachi F-4500 fluorescence spectrophotometer (Tokyo, Japan). The binding constant of the DHF–HSA complex was determined by fluorescence quenching, during which increasing amounts of DHF (final concentrations: 0, 0.5, 1.0, 2.0, 3.0, 4.0, 5.0, and 6.0 μM) were added to HSA (2 μM) in phosphate buffered saline (PBS; pH 7.4; containing 8.00 g/L NaCl, 0.20 g/L KCl, 1.81 g/L Na_2_HPO_4_ × 2H_2_O, and 0.24 g/L KH_2_PO_4_); then, emission spectra were recorded using 295-nm excitation wavelength (at 25 °C, in the presence of air). DHF-HSA interaction was evaluated using the graphical application of the Stern-Volmer equation [25,26]:

(1) $$\frac{I_{0}}{I}=1+K_{SV}+\left[Q\right],$$

where *I_0_* and *I* are the fluorescence emission intensity of HSA at 340 nm in the absence and presence of DHF, respectively. *K_SV_* denotes the Stern–Volmer quenching constant (unit: L/mol), and [*Q*] is the concentration of DHF (unit: mol/L). Furthermore, the binding constant (*K*; unit: L/mol) of the DHF–HSA complex was determined by nonlinear fitting applying the Hyperquad2006 software as described elsewhere [25,27].

The displacement of warfarin from HSA was investigated with the previously described fluorescence-based method [25,26]. Fluorescence emission spectra of warfarin and HSA (1.0 and 3.5 μM, respectively) were recorded in the presence of increasing amounts of DHF (final concentrations: 0.0, 0.5, 1.0, 2.0, 3.0, 5.0, and 10.0 μM) in PBS (pH 7.4), using 317 nm excitation wavelength. Emission signal of warfarin was evaluated at 379 nm.

Ultraviolet (UV)-Vis spectra of DHF were recorded employing a Halo DB-20 spectrophotometer (Dynamica; Dietikon, Switzerland), after which the inner filter effect of DHF was corrected in each experiment based on the following equation [26,28]:

(2) $$I_{corr}=I_{obs}\times e^{(A_{ex}+\;A_{em})/2},$$

where *I_corr_* and *I_obs_* indicate the corrected and the measured fluorescence emission intensity, respectively, while *A_ex_* and *A_em_* denote the absorption of DHF at the excitation and emission wavelengths used, respectively.

### 2.3. Ultrafiltratio 

To test the displacement of the Site I marker warfarin and the Site II marker naproxen from HSA by DHF, ultrafiltration experiments were performed, applying the previously described methods [29,30]. Pall Microsep Advance centrifugal devices with a 10-kDa molecular weight cut-off (MWCO) value (VWR; Budapest, Hungary) were applied. The samples contained warfarin and HSA (1.0 and 5.0 μM, respectively) or naproxen and HSA (1.0 and 1.5 μM, respectively) with and without DHF (10 or 20 μM). Before ultrafiltration, the filter units were washed once with 3.0 mL of distilled water and twice with 3.0 mL of PBS; then, samples (with a 2.5 mL volume) were driven through the filters by centrifugation (10 min, 7500× *g*, fixed-angle rotor, 25 °C). The concentrations of site markers in the filtrates were quantified with HPLC (see Section 2.5).

### 2.4. In Vitro Enzyme Assays

The inhibitory action of DHF on CYP2C9 was investigated as described earlier [25]. In the enzyme-catalyzed reaction, diclofenac is oxidized to 4′-hydroxydiclofenac. Racemic warfarin (at the same concentrations as DHF) was applied as a known inhibitor of CYP2C9 (positive control). The incubates (with 200 μL final volume) contained CypExpress 2C9 reagent (8 mg/mL) including also the NADPH (nicotinamide adenine dinucleotide phosphate, reduced) generating system, diclofenac (15 μM), and increasing concentrations of DHF (0, 15, 30, 45, and 60 μM) in potassium phosphate buffer (50 mM, pH 7.5). The incubation was carried out in a thermomixer (80 min, 700 rpm, 30 °C). The reaction was started by the addition of the enzyme and stopped by the addition of 100 μL of ice-cold methanol; then, the samples were centrifuged (10 min, 14,000× *g*, 25 °C). Diclofenac and 4′-hydroxydiclofenac were analyzed in the supernatants by HPLC (see Section 2.5).

To study the inhibitory effect of DHF on CYP2C19, *S*-mephenytoin was used as the substrate. During the enzyme-catalyzed reaction, 4-hydroxymephenytoin is formed. Ticlopidine, as the known inhibitor of CYP2C19, was employed as positive control (at the same concentrations as DHF). The incubates (with 200 μL final volume) contained CypExpress 2C19 reagent (15 mg/mL; containing also the NADPH generating system), *S*-mephenytoin (5 μM), NADP^+^ (200 μM), G6P (500 μM), and increasing concentrations of DHF (0, 5, 10, 15, and 20 μM) in potassium phosphate buffer (50 mM, pH 7.5). The incubation was performed in a thermomixer (120 min, 600 rpm, 30 °C). The reaction was started by the addition of the enzyme and stopped by the addition of 100 μL of ice-cold methanol. After centrifugation (10 min, 14,000× *g*, 25 °C), *S*-mephenytoin and 4-hydroxymephenytoin were quantified in the supernatants by HPLC (see Section 2.5).

The inhibitory effect of DHF on CYP3A4 was investigated as described previously [29]. In the enzyme-catalyzed reaction, testosterone is oxidized to 6β-hydroxytestosterone. Naringenin (at the same concentrations as DHF) was applied as the known inhibitor of CYP3A4 (positive control). The incubates (with 200 μL final volume) contained CypExpress 3A4 reagent (15 mg/mL; containing also the NADPH generating system), testosterone (5 μM), NADP^+^ (200 μM), G6P (500 μM), and increasing concentrations of DHF (0, 5, 10, 15, and 20 μM) in potassium phosphate buffer (50 mM, pH 7.5). Samples were incubated in thermomixer (180 min, 600 rpm, 30 °C). The reaction was started by the addition of the enzyme and stopped by the addition of 100 μL of ice-cold methanol. After centrifugation (10 min, 14,000× *g*, 25 °C), testosterone and 6β-hydroxytestosterone were analyzed in the supernatants by HPLC (see Section 2.5).

The inhibitory action of DHF on XO was investigated as described earlier [31]. In the enzyme-catalyzed reaction, 6-mercaptopurine is oxidized to 6-thiouric acid. Allopurinol (at the same concentrations as DHF) was applied as the known inhibitor of XO (positive control). The incubates (with 500 μL final volume) contained XO enzyme (0.01 unit/mL), 6-mercaptopurine (5 μM), and increasing concentrations of DHF (0, 5, 10, 15, and 20 μM) in sodium phosphate buffer (50 mM, pH 7.5). Samples were incubated in thermomixer (40 min, 700 rpm, 37 °C). The reaction was started by the addition of the enzyme and stopped by the addition of 30 μL of perchloric acid (6 M). After the samples were vortexed and centrifuged (5 min, 1,4000× *g*, room temperature), a 300-μL aliquot of the supernatant was transferred into another Eppendorf tube; then, 36 μL of potassium hydroxide (1 M) was added to this fraction. Samples were cooled to 3 °C, then, centrifuged (5 min, 14,000× *g*, 3°C). 6-mercaptopurine and 6-thiouric acid were quantified in the supernatants by HPLC (see Section 2.5).

### 2.5. HPLC Analyses

HPLC analyses applied a HPLC system equipped with a Waters 510 pump (Milford, MA, USA), a Rheodyne 7125 manual injector with a 20 μL sample loop, a Waters 486 UV-detector, and a Jasco FP-920 fluorescent detector (Tokyo, Japan). Chromatographic data were evaluated using Millenium Chromatography Manager Software (Waters; Milford, MA, USA).

The analysis of warfarin in the filtrates was performed applying the described method [29]. The samples were driven through a Nova-Pak C18 guard column (3.9 × 20 mm, 4 μm; Waters,) and a Nova-Pak C18 analytical column (3.9 × 150 mm, 4 μm; Waters). The mobile phase contained sodium phosphate buffer (25 mM, pH 7.0), methanol, and acetonitrile (70:25:5 *v/v*%). The isocratic elution was performed at 1.0 mL/min flow rate at room temperature. Warfarin was detected using 310 and 390 nm excitation and emission wavelengths, respectively.

Naproxen was analyzed in the filtrates as described earlier [29]. The samples were driven through a Security Guard (C18, 4.0 × 3.0 mm) guard column (Phenomenex; Torrance, CA, USA) linked to a Gemini C18 (150 × 4.6 mm, 3 μm; Phenomenex) analytical column. The mobile phase contained acetonitrile and sodium acetate buffer (6.9 mM, pH 4.0) (50:50 *v/v%*). The isocratic elution was performed at 1.0 mL/min flow rate at room temperature. Naproxen was detected at 230 nm.

Diclofenac and 4′-hydroxydiclofenac (CYP2C9 assay) were quantified as reported previously [25]. The samples were driven through a Phenomenex Security Guard (C8, 4.0 × 3.0 mm) guard column (Torrance, CA, USA) linked to an Eclipse C8 (150 × 4.6 mm, 5 μm; Agilent, Santa Clara, CA, USA) analytical column. The separation was carried out at 1.0 mL/min flow rate at room temperature. During the isocratic elution, the mobile phase contained acetonitrile and 6 mM orthophosphoric acid (52:48 *v/v*%). Diclofenac and 4′-hydroxydiclofenac were detected at 275 nm.

*S*-mephenytoin and 4-hydroxymephenytoin (CYP2C19 assay) were quantified employing a Phenomenex Security Guard (C8, 4.0 × 3.0 mm) guard column linked to a Phenomenex C8 (100 × 4.6 mm, 2.6 μm) analytical column. The mobile phase contained acetonitrile, methanol, and sodium phosphate buffer (10 mM, pH 4.55) (20:15:65 *v/v*%). The isocratic elution was performed at 1.0 mL/min flow rate at room temperature. *S*-mephenytoin and 4-hydroxymephenytoin were detected at 230 nm.

Testosterone and 6β-hydroxytestosterone (CYP3A4 assay) were quantified as reported previously [29]. The samples were driven through a Phenomenex Security Guard (C18, 4.0 × 3.0 mm) guard column linked to a Kinetex EVO C18 (150 × 4.6 mm, 5 μm) analytical column (Phenomenex). The mobile phase contained methanol, water, and glacial acetic acid (59:40:1 *v/v*%). The isocratic elution was performed at 1.2 mL/min flow rate at room temperature. Testosterone and 6β-hydroxytestosterone were detected at 240 nm.

6-mercaptopurine and 6-thiouric acid were quantified as described earlier [31]. The samples were driven through a Phenomenex SecurityGuard (C18, 4.0 × 3.0 mm) guard column linked to a Phenomenex Gemini-NX C18 (150 × 4.6 mm, 3 μm) analytical column. The eluent contained methanol, acetonitrile, and 0.02 M orthophosphoric acid (4:5:91 *v/v*%). The isocratic elution was performed at 0.8 mL/min flow rate at room temperature. 6-mercaptopurine and 6-thiouric acid were detected at 334 nm.

### 2.6. Statistics

Our plotted data indicate mean ± standard error of the mean (SEM) values derived from at least three independent experiments. Statistical evaluation of data was performed employing IBM SPSS Statistics software (Version 21; IBM Corporation, New York, NY, USA) using one-way ANOVA tests (*p* < 0.05 and *p* < 0.01).

## 3. Results

### 3.1. Interaction of DHF with HSA Based on Fluorescence Quenching Studies

**Figure 2:**
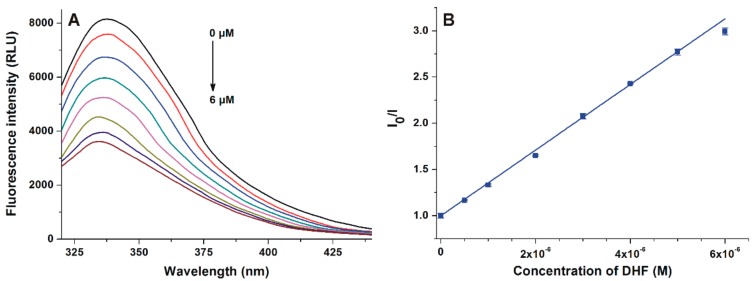
(**A**) Fluorescence emission spectrum of human serum albumin (HSA; 2 μM) in the presence of increasing concentrations (0.0, 0.5, 1.0, 2.0, 3.0, 4.0, 5.0, and 6.0 μM) of 7,8-dihydroxyflavone (DHF) in phosphate buffered saline (PBS; pH 7.4). (**B**) Stern–Volmer plot of DHF-HSA interaction (λ_ex_ = 295 nm, λ_em_ = 340 nm).

The complex formation of DHF with HSA was investigated using fluorescence quenching method. In a concentration-dependent fashion, DHF markedly decreased the emission signal of HSA at 340 nm (λ_ex_ = 295 nm; Figure 2A), and a slight blue shift of the emission maximum was also observed. After the inner-filter effect of DHF was corrected (see in Equation (2)), DHF-HSA interaction was evaluated applying the Stern–Volmer equation and the Hyperquad2006 software (see Section 2.2). The Stern–Volmer plot showed an excellent linearity (*R*^2^ = 0.998, *K_SV_* = 3.44 × 10^5^ ± 0.03 × 10^5^ L/mol; Figure 2B), and the binding constant was 4.87 × 10^5^ ± 0.07 × 10^5^ L/mol based on the nonlinear fitting with the Hyperquad2006 software.

### 3.2. Effects of DHF on the Fluorescence Signal of Warfarin–HSA Complex

**Figure 3:**
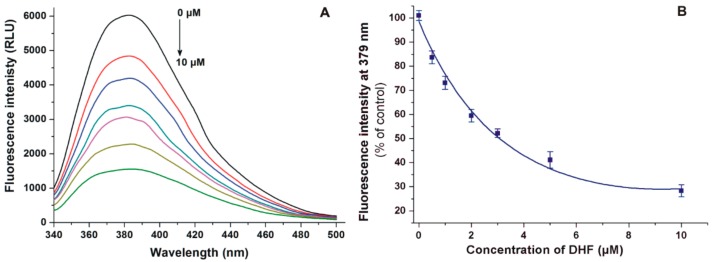
(**A**) Fluorescence emission spectrum of warfarin (1.0 μM) in the presence of HSA (3.5 μM) and increasing concentrations (0.0, 0.5, 1.0, 2.0, 3.0, 5.0, and 10.0 μM) of DHF in PBS (pH 7.4). (**B**) DHF induced decrease in the fluorescence emission of warfarin (λ_ex_ = 317 nm, λ_em_ = 379 nm).

First, the displacement of the Site I marker warfarin from HSA by DHF was investigated with a fluorescence spectroscopic model based on the principle that the HSA-bound warfarin shows markedly stronger fluorescence emission signal than the unbound molecule [25,32]. To test the displacing ability of DHF, increasing concentrations of the flavonoid were added to warfarin and HSA; then, the emission spectra were recorded (λ_ex_ = 317 nm). Even after the elimination of the inner-filter effect of DHF (see in Equation (2)), the flavonoid strongly decreased the fluorescence emission signal of warfarin at 379 nm in a concentration-dependent fashion (Figure 3).

### 3.3. Ultrafiltration Studies

**Figure 4:**
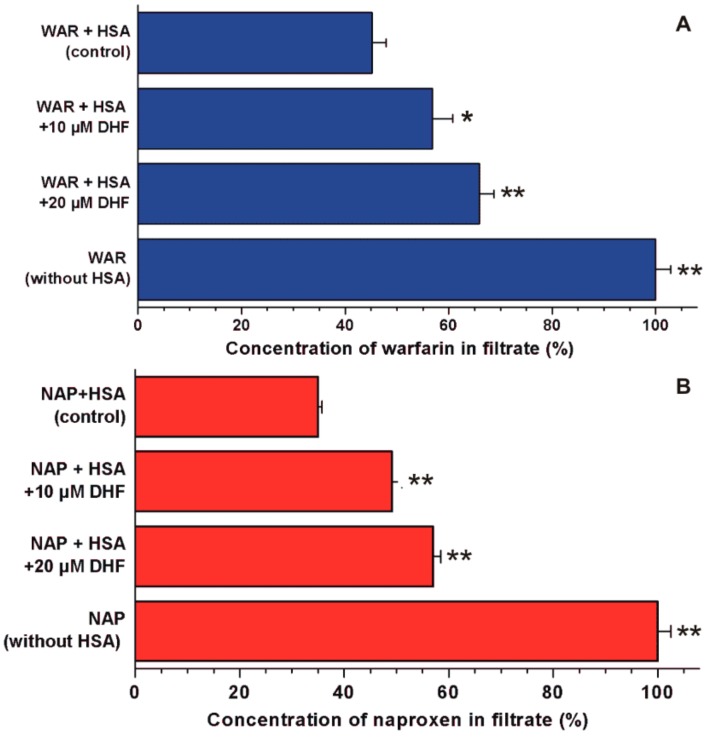
Displacement of Site I (warfarin, WAR) and Site II (naproxen, NAP) markers from HSA by DHF. Concentrations of warfarin (**A**) and naproxen (**B**) in the filtrate: Before the ultrafiltration, the samples contained warfarin and HSA (1.0 and 5.0 μM, respectively) or naproxen and HSA (1.0 and 1.5 μM, respectively) with or without 10 and 20 μM DHF in PBS (pH 7.4; * *p* < 0.05, ** *p* < 0.01). In each model, the filtered concentration of site markers was compared to the concentration measured in the filtrate when no HSA was added to the site markers (100%).

In the following experiments, the displacement of the Site I marker warfarin and the Site II marker naproxen by DHF was investigated employing ultrafiltration. Since HSA and albumin-bound molecules cannot pass through the filter unit with a 10-kDa MWCO value, the increased concentration of site markers in the filtrate suggests their displacement from albumin by the test compound [29,30]. In a concentration-dependent fashion, DHF markedly increased the concentrations of both site markers in the filtrates (Figure 4). The 12 and 21% fractions of total warfarin became unbound in the presence of 10 and 20 μM DHF, respectively. In addition, the appearance of further 14 and 22% of total naproxen was observed in the filtrate, induced by 10 and 20 μM of DHF, respectively.

### 3.4. Inhibition of Biotransformation Enzymes by DHF

**Figure 5:**
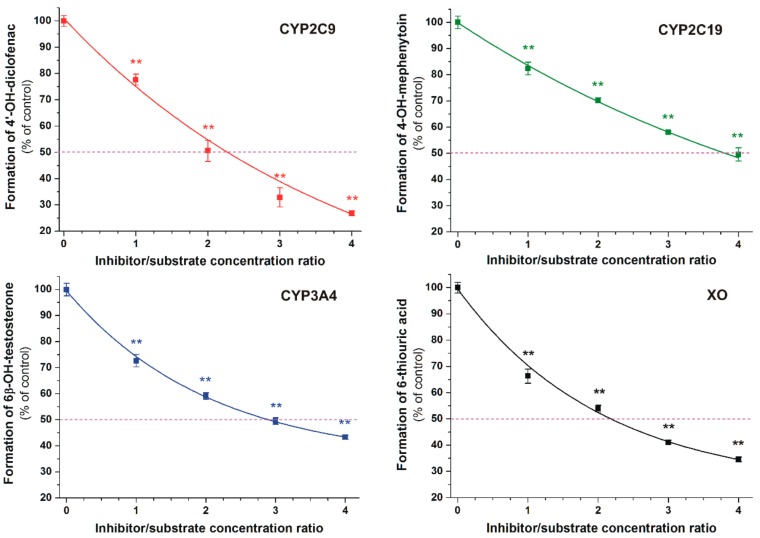
Concentration-dependent inhibitory effects of DHF on CYP2C9, CYP2C19, CYP3A4, and xanthine oxidase (XO) enzymes (** *p* < 0.01). The 50% inhibition of metabolite formation is marked with the violet dashed line.

Inhibition of CYP2C9, CYP2C19, CYP3A4, and XO enzymes was tested applying in vitro enzyme assays. DHF significantly inhibited the metabolite formation in each enzyme assay, even at equimolar concentrations with the substrates (Figure 5). The absolute and relative IC_50_ values (the inhibitor concentration which induces 50% decreases in metabolite formation) of DHF and the corresponding positive controls as well as the α values (IC_50_ of DHF divided by the IC_50_ value of the positive control) are listed in Table 1. DHF induced 50% inhibition at approximately two-fold concentration vs. the substrates in CYP2C9 and XO assays. Furthermore, the IC_50_ values of DHF were circa three- and four-fold higher compared to the substrate concentrations in the CYP3A4 and CYP2C19 assays, respectively. The flavonoid proved to be a similarly potent inhibitor of CYP2C9 to the positive control warfarin as well as 1.5- to 1.9-fold weaker inhibitor of other enzymes tested (CYP2C19, CYP3A4, and XO) vs. the corresponding positive controls.

## 4. Discussion

DHF is a promising candidate in the treatment of central nervous system diseases; however, its possible pharmacokinetic interactions with serum albumin and biotransformation enzymes have not been characterized. Therefore, in this study, we aimed to examine the interaction of DHF with serum albumin employing fluorescence spectroscopy and ultrafiltration, and the inhibitory effect of DHF on CYP and XO enzymes was also tested using in vitro enzyme assays.

In quenching studies, after the correction of the inner filter effect, the 0.5 μM and higher concentrations of DHF (vs. 2 μM HSA) strongly decreased the fluorescence emission signal of albumin at 340 nm (Figure 2). This observation suggests the formation of a stable DHF–albumin complex. The single tryptophan residue (Trp-214) of HSA (which is largely responsible for the intrinsic fluorescence of the protein) is located in subdomain IIA [33,34]. Therefore, the strong quenching effect of DHF on HSA suggests that the binding site of DHF needs to be near to the Trp-214 moiety. Furthermore, in the presence of DHF, a blue shift in the fluorescence emission spectrum of HSA was observed (Figure 2). It can be likely explained by the interaction of DHF with HSA, which leads to the increased lipophilicity of the microenvironment around the Trp-214 residue [35]. Furthermore, the binding of DHF strongly decreases the fluorescence of Trp-214, resulting in the more dominant fluorescence signal of tyrosine amino acids vs. the sole tryptophan molecule [36]. It may also be responsible for the blue shift in the fluorescence emission spectrum of albumin. Both linear and nonlinear fittings (received with the Stern–Volmer equation and the Hyperquad2006 software, respectively) showed good correlation with the 1:1 stoichiometry of complex formation. Our results demonstrate that DHF binds to HSA with two-fold higher affinity compared to chrysin (5,7-dihydroxyflavone, a structural isomer of DHF; *K* = 3.4 × 10^5^ L/mol) [37] and the Site I marker warfarin (*K* = 3.4 × 10^5^ L/mol) [32], which suggests the potential biological importance of DHF–HSA interaction. Nevertheless, it is important to note that the plasma concentrations of DHF seem to be relatively low (33–192 nM) based on animal studies (after the per os administration of 15–50 mg/kg DHF to mice) [14].

DHF markedly decreased the emission signal of warfarin at 379 nm (Figure 3). Since the inner filter effect was corrected, this observation suggests the displacement of warfarin from HSA because HSA-bound warfarin exerts much higher fluorescence vs. the unbound fluorophore [25,32]. Based on the previous investigations using the same model, the displacing ability of DHF vs. warfarin seems similar to chrysin [37], quercetin [25], and diosmetin [26]. The ability of flavonoids to displace warfarin from albumin has been reported in several studies [26,32,38,39]; however, Rimac et al. [40] suggest the potential cooperative binding of warfarin and some flavonoids. Therefore, the displacement of warfarin may be resulted from competitive and/or allosteric interactions, as it was also suggested regarding diosmetin [26]. In ultrafiltration studies, DHF caused significant increases in the concentrations of both Site I and II markers in the filtrate (Figure 4), showing that DHF can displace both Site I and Site II ligands from HSA. Under the same experimental conditions, diosmetin showed a similar displacing ability vs. warfarin than DHF [26] while quercetin showed considerably stronger effect compared to both DHF and diosmetin [25]. At 20 μM concentration, chrysin displaced a similar fraction of warfarin and naproxen than DHF in the same models [37]. The significant displacement of highly albumin-bound drugs from HSA causes the strong elevation of their free fraction in the circulation, leading to their increased tissue uptake or faster elimination [16,17]. If high concentrations of DHF appear in circulation, it may be able to displace Site I or Site II ligands. However, based on our current knowledge regarding the plasma concentrations and displacing ability of DHF, it does not seem likely.

In each enzyme assay, DHF significantly decreased metabolite formation (Figure 5 and Table 1), suggesting that DHF may interfere with the biotransformation of several medications. DHF proved to be a similarly potent inhibitor of the CYP2C9-catalyzed 4′-hydroxydiclofenac formation to warfarin (Table 1). Racemic warfarin was applied in our experiments as the positive control. The (*S*)-warfarin is the substrate of CYP2C9 enzyme; nevertheless, 7-hydroxywarfarin (formed via the CYP2C9-catalyzed hydroxylation) can also inhibit the enzyme [41]. Inhibition of CYP2C9 by some other flavonoids has been also described previously, including 6-hydroxyflavone, quercetin, apigenin, luteolin, casticin, and diosmetin [26,29,42]. In the same model applied in this study, casticin was 1.3-fold weaker, while diosmetin was 2.7-fold stronger inhibitor of CYP2C9 enzyme compared to DHF [26,29]. Despite the fact that quercetin and its metabolites proved to be weak inhibitors of CYP2C9-catalyzed diclofenac hydroxylation [25], quercetin (500 mg, administered twice daily) significantly inhibited the metabolism of diclofenac in healthy human volunteers [43]. These observations suggest the potential hazardous consequences of the simultaneous administration of DHF with certain drugs eliminated through CYP2C9.

DHF inhibited the CYP2C19-catalyzed 4′-hydroxymephenytoin formation 1.8-fold weaker than ticlopidine. Among the previously tested flavonoids, quercetin showed significant inhibitory effect on CYP2C19 (using human liver microsomes and *S*-mephenytoin as substrate) [44,45]. The oral antiplatelet drug ticlopidine (a competitive inhibitor of CYP2C19) reaches from 1.3 to 2.6 μM peak plasma concentration after the oral administration of a single 250 mg dose [46]. Furthermore, during chronic treatment with ticlopidine (oral administration of 250 mg twice daily), its peak plasma concentrations were ranging from 3.4 to 4.1 μM in men [46]. The coadministration of ticlopidine with phenytoin led to phenytoin intoxication in an epileptic patient [47]. In addition, ticlopidine (administered orally 3 × 100 mg daily; average peak plasma concentrations: approximately 3.5 μM) increased the serum concentrations of omeprazole in healthy subjects due to the inhibition of its CYP2C19-mediated metabolism [48]. Considering the previous data that even low micromolar plasma concentrations of ticlopidine can induce clinically relevant CYP2C19 inhibition as well as our observation that DHF is a slightly weaker inhibitor of CYP2C19 than ticlopidine, it seems possible that DHF may interfere with the CYP2C19-mediated elimination of some drugs.

DHF inhibited the CYP3A4-catalyzed 6β-hydroxytestosterone formation 1.4-fold weaker than the positive control naringenin (Table 1). Using the same model described here, resveratrol was a two-fold stronger inhibitor of CYP3A4 while casticin has similar inhibitory effect on this enzyme compared to DHF [29]. Furthermore, in in vitro studies, chrysin also proved to be a potent inhibitor of CYP3A4 [49]. CYP3A4 is the most abundant cytochrome enzyme in enterocytes and hepatocytes; its inhibition can lead to the development of several clinically relevant food–drug and drug–drug interactions [50,51,52]. Hypericin, hyperforin, and I3,II8-biapigenin in St John’s wort (*Hypericum perforatum,* Hypericaceae) and naringenin, bergamottin, and 6,7-dihydroxybergamottin in grapefruit (*Citrus paradisi,* Rutaceae) juice can strongly interfere with the CYP3A4-mediated biotransformation of drugs (e.g., some statins) [53]. These data show that high intake of DHF may interfere with CYP3A4-mediated elimination of some drugs.

DHF inhibited the XO-catalyzed 6-thiouric acid formation 1.7-fold weaker than the positive control allopurinol (Table 1). It is well known that flavonoid aglycones (including chrysin and quercetin) are strong inhibitors of XO-catalyzed xanthine oxidation [7,54]; however, we have limited data regarding the effects of flavonoids on 6-mercatopurine oxidation. A recent study demonstrated that quercetin and its sulfate or methyl conjugates are similarly strong inhibitors of XO-catalyzed xanthine and 6-mercaptopurine oxidation and are approximately ten-fold stronger inhibitors of 6-mercaptopurine oxidation than allopurinol [31]. Inhibition of XO-mediated inactivation of the antitumor agent 6-mercaptopurine by allopurinol can result in severe or even fatal consequences [55]. However, the therapeutic plasma concentrations of allopurinol and its metabolite oxipurinol (both are active inhibitors of XO) are tens of micromoles [31,56]. Therefore, it is unlikely that DHF can reach similar plasma and tissue concentrations to allopurinol/oxipurinol, which makes less likely the development of clinically relevant pharmacokinetic interaction of DHF with 6-mercaptopurine.

## 5. Conclusions

The application of flavonoids as drugs and/or dietary supplements is emerging. Many studies suggest that flavonoids may be able to interfere with the pharmacokinetics of certain drugs due to their interactions with serum albumin, biotransformation enzymes, and drug transporters. However, commonly limited information is available regarding the pharmacokinetic interactions of an individual flavonoid and/or its metabolites, and the clinical relevance of these interactions is usually unclear. Our results demonstrate that DHF forms a stable complex with HSA and it is able to displace both Site I and Site II ligands. Furthermore, DHF can strongly inhibit each enzyme tested. Inhibition of CYP2C9 enzyme seems the most relevant; however, the effects of DHF on CYP2C19 and CYP3A4 may also cause the development of pharmacokinetic interactions with some medications. Based on our observations, the simultaneous administration of DHF with drugs should be carefully considered. However, further studies seem reasonable to explore the in vivo relevance of our in vitro results.

## Figures and Tables

**Table 1:** IC_50_, relative IC_50_, and α values of DHF regarding the four tested biotransformation enzymes. IC_50_ denotes the concentration of DHF which induces 50% inhibition of the metabolite formation, IC_50(rel)_ is the IC_50_ of DHF divided by the substrate concentration applied, while α marks the IC_50_ of DHF divided by the IC_50_ value of the corresponding positive control.

Enzyme	Substrate Concentration (μM)	Inhibitor	IC_50_ (μM)	IC_50(rel)_	α
CYP2C9	15	DHF	34.5	2.3	1.0
		WAR (positive ctrl)	34.5	2.3	
CYP2C19	5.0	DHF	19.0	3.8	1.9
		TIC (positive ctrl)	10.0	2.0	
CYP3A4	5.0	DHF	14.5	2.9	1.5
		NAR (positive ctrl)	10.0	2.0	
XO	5.0	DHF	11.0	2.2	1.6
		APU (positive ctrl)	7.0	1.4	

WAR, warfarin; TIC, ticlopidine; NAR, naringenin; APU, allopurinol
